# Investigation of biomaterials by human epithelial gingiva cells: an *in vitro* study

**DOI:** 10.1186/1746-160X-8-35

**Published:** 2012-12-15

**Authors:** Jörg Neunzehn, Beate Lüttenberg, Hans-Peter Wiesmann

**Affiliations:** 1 Max Bergmann Center of Biomaterials and Institute of Materials Science, Technische Universität Dresden, Budapester Str. 27, Dresden, D-01069, Germany; 2 Department for Cranio- and Maxillofacial Surgery, Westfälische-Wilhelms-Universität Münster, Albert-Schweitzer-Campus 1, Gebäude: W30, Münster, D-48149, Germany

**Keywords:** Human gingiva, Epithel, Biomaterials, Keratinocytes, *In vitro* study

## Abstract

**Introduction:**

In modern medicine and dentistry the use of biomaterials is a fast developing field of increasing interest. Especially in dentistry the interaction between biomaterials like implant materials and the soft tissue in the oral cavity is in the focus of daily research. In this context the high importance of testing materials and their surfaces concerning their biocompatibility towards corresponding cells is very likely. For this purpose this study investigates cells derived from human gingival biopsies on different materials and surfaces.

**Methods:**

Cells in this study were cultivated out of human biopsies by a grow out explant technique and were sub cultivated on titanium, zirconium dioxide and collagen membrane specimens. To characterise the cells on the material surfaces used in this study immunohistochemical and histological staining techniques as well as different methods of microscopy (light microscopy and SEM) were applied.

**Results:**

With the aid of the explant technique and the chosen cell cultivation method it was possible to investigate the human gingiva derived cells on different materials. The data of the present study show that the human gingival cells attach and proliferate on all three tested materials by exhibiting characteristic gingival keratinocyte protein expression even after long periods of culture e.g. up to 70 days.

**Conclusions:**

It could be shown that the three tested materials titanium, zirconium dioxide and collagen membrane (and their special surfaces) are good candidates for the application as materials in the dental gingival environment or, in the case of the collagen membrane as scaffold/cell-carrier for human gingival cells in tissue engineering.

## Introduction

Over the last two decades, no other area of modern dentistry developed as fast as implantology. Due to the therapeutic opportunities of osseointegrative implants, the dental implants have become a reliable tool in modern dentistry. For the clinical success of dental implants several factors are crucial. In addition to osseointegration a successful growth and healing of the soft tissue in the oral cavity round the implant is an important criterion for the long-term success of an implant.

As it is known that the surface of the implant has direct influence on the osseointegration process, surface structures are one field of intensive research. A large number of different surface treatments can be applied to alter surface topography of titanium implants, including machining/micromachining, particle blasting, titan plasma spraying, HA plasma spraying, chemical/electrochemical etching and anodization. The topographic features that are obtained on the implant surface can range from nanometres to millimetres, i.e. from lower cell-size scale to tissue scale.

The attachment and interaction of all the involved cells (like osteoblasts, fibroblasts or epithelial cells) are important phenomena in clinical implant dentistry. One major consideration in manufacturing implants is to produce surfaces and materials that promote the expected and requested responses in the directly affected cells and the surrounding tissues [1-3].

In addition to osseointegration, a complication-free healing of the gingival soft tissue is very important to achieve a long-term success. Surface characteristics and composition of the implants are also responsible for soft tissue attachment and function. Additionally various postoperative methods are used to provide the recovery of the soft tissue to assure both satisfying aesthetic outcomes as well as the successful insertion of the implant in the soft tissue and a long storage period.

Nevertheless, biological failures happen and can hinder the integration of an implant in the bony tissue storage and/or the soft tissue border. A sufficiently large area of fixed, keratinized mucosa benefits the successful healing of the implant as a barrier against mechanical effects of the lips and cheek muscles. It also functions as a treshold and builds up a protective defend against infections caused by microorganisms or other inflammatory agents coming from the outside. Tissue inflammation with peri-implantitis and bone loss as a possible consequence can in the end lead to the loss of an implant.

For testing biomaterials and/or cell reactions towards materials and surfaces in dental implantology and tissue regeneration different studies with different cell types were performed over the last years [4-7]. A lot of tests and studies dealing with the nature of implant surfaces have shown that different cells also behave differently towards various materials and surface structures and modifications [8-12]. Although *in vitro* studies can not reflect the *in vivo* situation in all its complexity, *in vitro* experiments give a first impression of probable reactions that might also occur in the clinical situation and they allows studies under defined and more limited conditions.

On the basis of other investigations concerning the cultivation of human gingival keratinocytes [13-15] one aim of this work was to apply a successful isolation method and to characterize the human epithelial cells being obtained by the gingival biopsies.

Additionally the present study used these gingival derived cells to test different materials of modern dentistry, e.g. for dental implants, prosthetics and scaffolds in tissue engineering, concerning their biocompatibility.

## Material and methods

### Tissue collection

The gingival tissue samples used in this study were obtained from 15 healthy adult patients undergoing dental surgery at the oral and maxillofacial surgery department at Münster University Hospital. The study was approved by the Ethics Committee of the Münster University Hospital.

### Cell culture

Gingival cells were derived from human gingival biopsies. On the basis of 29 biopsies from 15 different patients, 242 samples were prepared to be used in this study.

After explantation, the tissue samples were washed in PBS and culture media (see below). The soft, jelly-like connective tissue of the samples was separated from the epithelial part by a scalpel. The resulting harder, epithelial parts were cut into thin slices (approximately 0.5 mm) and placed into cell culture dishes with the cutting area facing down. The tissue was left to adhere to the culture dishes for 10 min, DMEM (Biochrom) supplemented with 10% of FCS (Biochrom), 1% penicillin/streptomycin (Biochrom), 1% amphotericin B (Biochrom), 1% L-glutamine (Biochrom), 1 mg/l hydrocortisone (Biochrom), 5 mg/l insulin (Biochrom), 10 μg/l EGF (Biomol,) and 10 μg/l cholera toxin (Sigma) was added and tissue parts were incubated at 37°C and 5% CO_2_ in humidified air. Contamination and proliferation of the cell cultures were examined daily by inverted light microscopy.

Fibroblasts, shown as spindle shaped cells, were mechanically removed by sucking.

After reaching confluence (15 to 21 days), the cells were detached using 0.25% trypsin in ethylenediaminetetraacetat (EDTA) (diluted with PBS; 1:1; Invitrogen/Gibco). After detachment the cells were centrifuged, resuspended and counted (Casy Modell TT, Schärfe System, Germany). Then, the cells were sub cultivated on polystyrene culture dishes and on different material specimens, respectively.

### Titanium/Zirconium dioxide

The specimens used, were nano structured titanium (or more properly titanium oxide) and polycrystalline zirconium dioxide (ZrO_2_). The titanium specimens were 13.0 mm in diameter and 2.0 mm in thickness. The zirconia was a disc-like specimen with a diameter of 11.0 mm and a thickness of 1.0 mm. Specimens were sterilized for one hour in 70% ethanol before cells were seeded.

Specimens were cleaned by a mixture of 0.1 M tris (pH 7.4) and 0.1% SDS (Sodium Dodecyl Sulfate) in distilled water and scrubbed with cotton pellets. After thoroughly rinsing with distilled water the specimens were dried and stored.

### Collagen

Resodont (Resodont, RESORBA, Germany) is a white, compressed and tear-resistant membrane. It is made up of equine collagen fibrils and can be absorbed by the tissue. This material is often used for guided bone regeneration and is known to support the regeneration of periodontal tissue.

For cell cultivation the membrane was cut into squares with 0.9 mm in length, put into fitting chamber slides, was washed twice with culture medium and stored at 37°C and 5% CO_2_ for later use.

### Cell sub cultivation on materials

To cultivate the cells on the different materials, the tissue pieces were removed after 15–20 days. The confluent primary gingiva cells were detached by the use of trypsin (0.25% trypsin/EDTA, diluted with PBS; 1:1) for 10 min at 37°C. Cells were centrifuged (FUNCTIONline, Heraeus, Germany, 1200 × *g*, 5 min, 20°C), resuspended in complete fresh medium and counted (Casy Modell TT, Schärfe System, Germany).

Cells were seeded on the titanium and ZrO_2_ samples with 6 × 10^4^ cells/cm^2^. The same was done with the collagen specimens and with 15 × 10^5^ cells/cm^2^ respectively.

Sub cultivation of the cells was done on polystyrene cell culture dishes as control.

### Richardson staining

Richardson staining was accomplished with a blue dye (Methylen blue Azur II). Cells were methanol-fixed. After decanting the methanol 2–3 drops of the freshly mixed warm solution were applied and cells were incubated for 2 min at 60°C. Finally, the specimens were rinsed with distilled water, taken out of the wells, rinsed again to remove any excess stain and left to dry upside-down in a dark dry chamber.

### Haematoxylin-Eosin-staining (HE)

Cells on the specimens were methanol fixed and stained by haematoxylin and eosin. Haematoxylin dyes the nuclei blue and afterwards the connective tissue was marked pink by the use of eosin. The samples were treated by an ascending alcohol series (50%, 70%, 96% and 100%) for differentiation and dehydration. After this procedure the samples dried at room temperature.

### Immunohistochemistry

Cells were characterized by using the primary antibodies cytokeratin anti-human mouse monoclonal Clones [AE1/AE3] (Dako Code M3515; binding the cytokeratins 1,2,3,4,5,6,7,8,10,13,14,15,16,19; working dilution: 1:50) and p63 antibody [4A4] mouse monoclonal (Gene Tex, Inc.; Cat:No.: GTX23239; working dilution: 1:60). p63 is highly expressed in the basal layers of many epithelial tissues and human epidermal stem cells. A very low expression of this marker is shown by partially differentiated cells.

For immunohistochemistry staining the culture medium was removed, cells were washed three times with TBST (tris buffered saline with Tween) for 5 min and fixed for 20 min at −20°C with methanol. Cells were blocked in blocking solution (Candor Bioscience Germany) for 15 min at 37°C and incubated with the primary antibody (in blocking solution) for one hour at 37°C respectively.

Afterwards, specimens were washed three times with TBST and incubated with the secondary antibody (Dako-Cytomation, EnVision + −System, Labelled Polymer-HRP, Anti-Mouse, Dako; Alexa Fluor 546, Anti-Mouse, Abcam; diluted 1:100 with blocking solution) for 1 hour at 37°C.

After washing twice with TBST for 5 min the culture dishes were left upside-down to dry in the dark. Labelled cells were examined by (fluorescence)-light-microscopy.

### Paraffin embedding

To investigate the cells on the collagen membrane (Resodont) the samples were fixed with 4% formalin for 24 h. After washing for 45 min the samples were dehydrated by an ascending alcohol series. Subsequently, the specimens were embedded in paraffin after being treated for 60 min twice with methylbenzoat and benzol.

The paraffin blocks were cut into slices (thickness 3–4 μm) and drawn up on microscope slides. After paraffin removal by xylol treatment and a descending alcohol series the cells on paraffin free collagen slices were also analysed by immunohistochemistry staining using the anti-cytokeratin primary antibody (see above).

### Scanning electron microscopy

For cell fixation the medium was removed and the specimens were washed three times with TBST for 5 min and RT (room temperature), and then fixed with glutaraldehyde. To dehydrate the cells a series of ascending alcohol was used. At RT the specimens were immersed in 90% Ethanol, 96% Isopropanol and 100% Isopropanol for 20 min respectively.

For the titanium and zirconium dioxide samples critical point drying (CPD) was done with CPD 010 (Balzers Union, Germany).

The two implant material surfaces were also investigated by energy disperse x-ray analysis (EDX).

## Results

### Tissue preparation, culture development and sub culturing

After the successful preparation and separation of epithelium from the connective tissue, the average number of days required for the cells to grow and migrate out of the gingival tissue origin was 3 days.

224 samples of the 242 tissue samples showed outgrowing of epithelial cells. This corresponds to a success rate from the direct explant technique of about 93%. In the cultures of 18 samples (7%) no cell growth could be detected for unknown reason. In the case of 116 samples even an excrescence success rate of 100% could be reached within this study. None of the samples showed microbial infections or other noxious factors.

After 3 days the first epithelial cells appeared in the boundary area of the gingival explants. These first out grown cells were low-contrasted and seemed to be partly transparent. 24 hours later these cells showed a more contrasted phenotypic structure. The cells built up a closed cell layer on the polystyrene substrate after 7 days. After 14 to 21 days the cells attained nearly confluence, depending on the dish diameter and the amount of samples initially used. During this process the shape of the cells changed from small oval-spherical with a narrow seam of cytoplasm around the nucleus to bigger polygonal cells with a larger cytoplasm ring surrounding the nucleus. (Sub-) cultivation of the cells was possible over a period of time up to 70 days.

### Specimen material

Macroscopically both surfaces of the investigated titanium and zirconia samples appeared to be smooth. To get more detailed information, the topographical characteristics and the composition of the two implant surfaces were investigated by scanning electron microscopy and energy disperse x-ray analysis.

### Scanning electron microscopic imaging of the specimens

**Figure 1:**
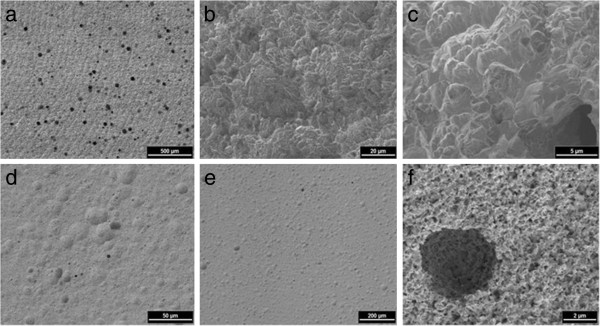
**SEM of Titanium and Zirconium dioxide specimen material.** Titanium specimen: co-centric microgrooves (**a**), topographical appearance (**b**), magnification (**c**). Zirconium dioxide: Faint co-centric micro grooved surface (**d**), topographical appearance (**e**), micro pore (**f**).

The titanium surface (Figure 1a-c) showed a homogeneous distribution of closed micro pores steadily increasing in number towards the circumference of the specimen ranging from 5–50 μm in diameter. Shallow co-centric microgrooves with regular increase in diameter are also visible. The zirconium dioxide specimens (Figure 1d-f) showed a smoother surface with fewer pores. They seemed to be not as deep as those in the titanium specimens and were more scarcely distributed. Furthermore, the surface exhibited faintly micro grooved structure with a similar distribution as the titanium specimens. Both surfaces showed nano scaled structures between these micro grooves.

### Energy disperse x-ray analysis (EDX) of the specimen materials

**Figure 2:**
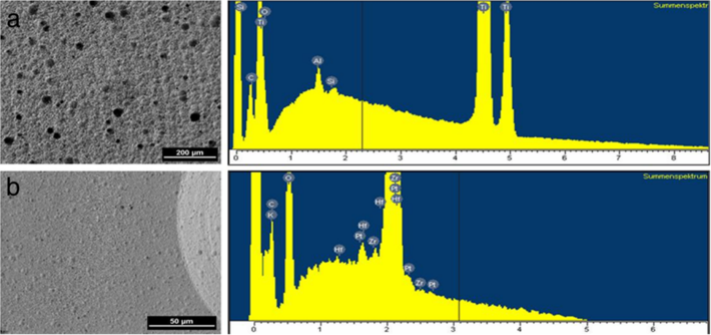
EDX-spectres of the titanium (a) and zirconium dioxide specimens (b).

The EDX analysis of the titanium specimens clearly showed the presence of the elements titanium and oxygen, additionally also silicon, aluminium and carbon were detected (Figure 2a). The zirconia specimens showed the presence of zirconium and oxygen and the elements hafnium, aluminium, potassium, carbon and platinum (Figure 2b). The specimens were rendered conductive by means of a carbon/platinum coating. The results of the EDX analysis are shown in Figure 2.

### Cell characterisation

Cells were methanol fixed and stained either with haematoxylin/eosin (HE) or the Richardson method. Further characterisation of the cells in culture was carried out by the use of two different specific epithelial markers (cytokeratin anti-human, p63-antibody) by applying immunhistochemistry. 

Before sub cultivation

**Figure 3:**
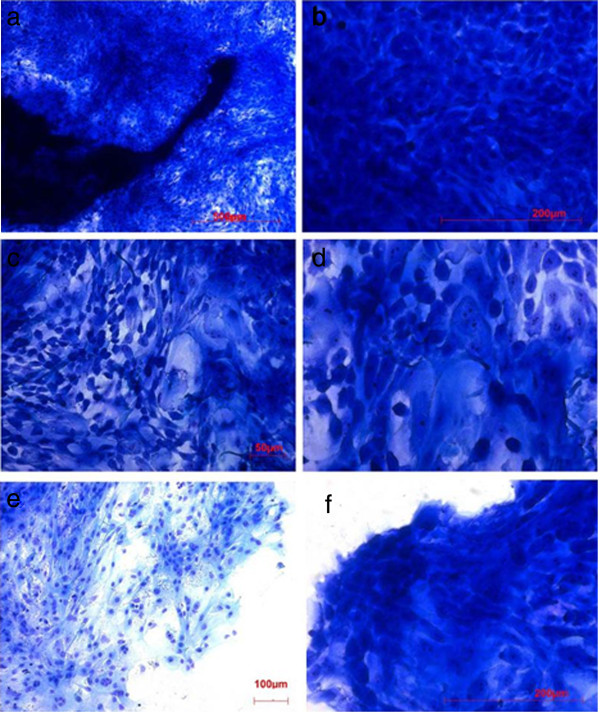
Richardson-staining of the out grown cells.

The results of both the HE- (data not shown) and the Richardson-staining (Figure 3) showed huge variations of the cells regarding their size, the size-ratio between cell nucleus and the cytoplasm and cell-cell-contacts. This variation in shape and size of the epithelial cells seemed to be independent from the period of cultivation (Figure 3).

Next to the tissue samples cells were small with narrow seam of cytoplasm around the nucleus (Figure 3a, b). These round or sometimes polygonal cells appeared close together with partial overlapping. This small cell type was not only found in the immediate vicinity of the tissue pieces, but also in the margins and within the cell layers. The cell nuclei differed, regardless of their position within the cell layer (Figure 3c, d).

Especially at the edge of the cell layers a wide range of variations of the cell-phenotypes could be observed (Figure 3e, f).

In addition to the histological investigations immunohistochemistry staining of the cells on the different materials were done. To identify the out grown cells as epithelial cells independent from their phenotype an antibody against a variety of human cytokeratins (see materials and methods), an ideally suited marker for the characterization of keratinocytes was used. Virtually all cells reacted positive to this typical epithelial marker. 

After sub cultivation

**Figure 4:**
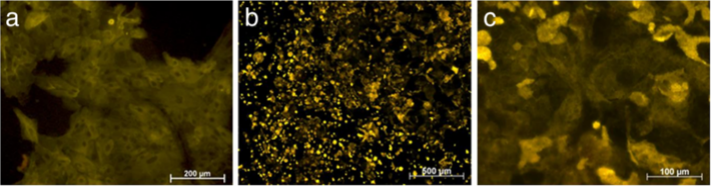
**Cultured epithelial cells after passage.** cytokeratin immunohistochemistry on polystyrene (**a**), cytokeratin immunohistochemistry on titanium after 2 weeks (**b** + **c**).

**Figure 5:**
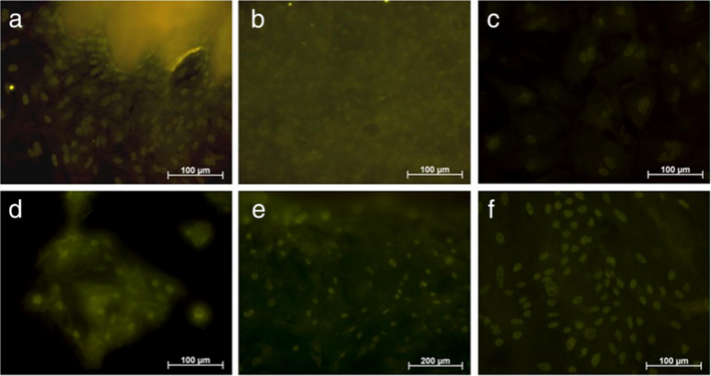
**Immunohistochemistry staining with anti-p63 on polystyrene.** Tissue with outgrown cells after 3 days (**a**), multi-layered cells after 28 days (**b**), positively stained cells after 70 days (**c**), 1 day (**d**), 6 days (**e**) and 10 days after sub cultivation (**f**).

Two weeks after sub cultivation the cells were seeded onto polystyrene and the titanium surface. It could be shown that the gingival cells attached to these two materials after sub cultivation. The immunohistochemical staining done with the cultured cells on the different materials demonstrated their epithelial character (Figure 4). This was also stressed by using p63-antibody, a homologue of the tumor suppressor gene product p53, which additionally characterized the sub-cultivated cells as gingival keratinocytes. Almost all cells e.g. cultured on polystyrene could be detected by using the p63-antibody (Figure 5).

### Specific cell behaviour/cell attachment

During cell cultivation it could be seen that the cells built up cell associations, a typical attribute of epithelial cells. The development of cell associations could also be observed after sub culturing on polystyrene and the other materials. The cells on polystyrene were equally distributed on the material surface already one day after seeding. After 6 and 14 days cells started to interact and built up united cell structures. These agglomerated cells were also seen after 8 days of the second sub cultivation.

**Figure 6:**
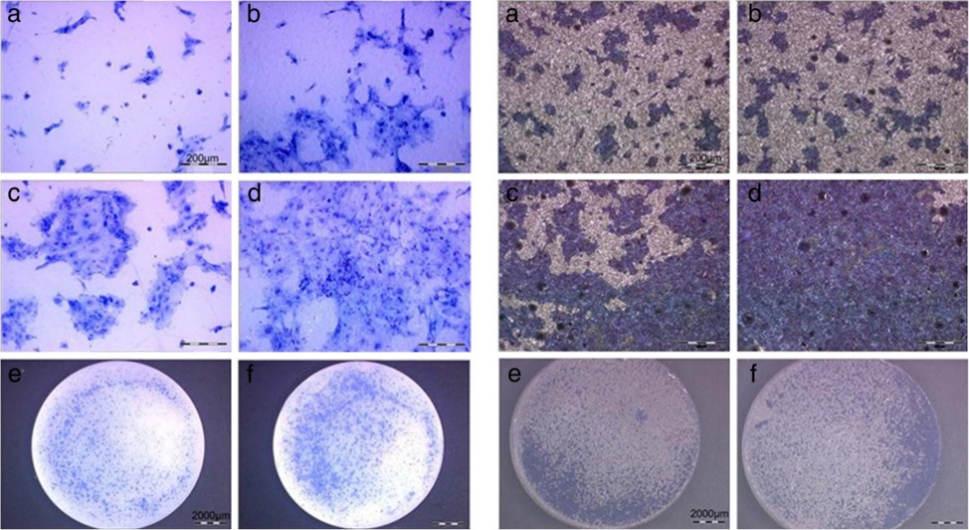
**Sub cultivated cells on zirconium dioxide (left side) and titanium (right side) (60. 000 cells/cm**^**2**^**after 24 hours (a, c, e) and 48 hours (b, d, f).**

To demonstrate the characteristics of the cells after shorter periods on the two implant materials 60.000 cells/cm^2^ were cultivated on the material samples for 24 and 48 hours. After staining, the cells on the material, surfaces were investigated by light microscopy. In both cases after 24–48 hours cell layers were observed (Figure 6).

**Figure 7:**
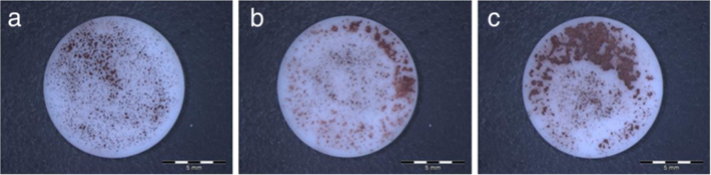
Anti-cytokeratin detected cells after 1 (a), 7 (b), 14 (c) days on ZrO_2_.

In addition to the Richardson staining the cells on the ZrO_2_ samples were also detected by immunohistochemistry with anti-cytokeratin after different periods of time (Figure 7) to reprove the epithelial character.

**Figure 8:**
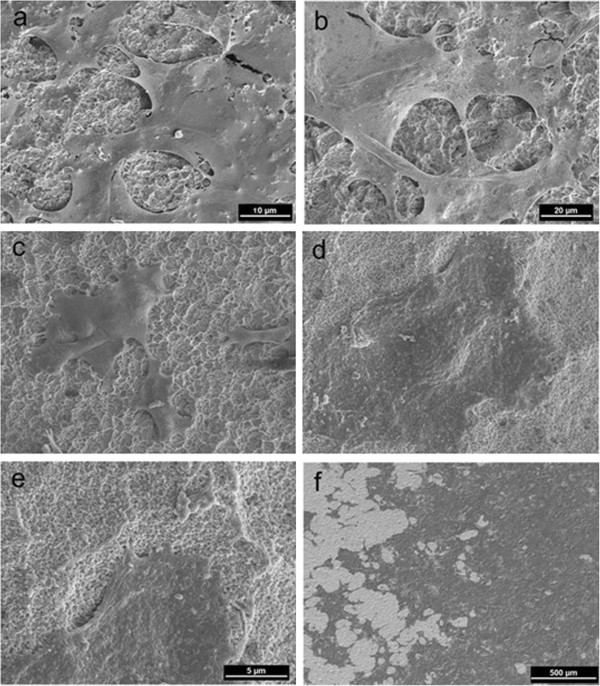
SEM-pictures: Cells on Titanium (a, b, c) and on zirconium dioxide (d, e) after 24 h and on zirconium dioxide after 48 h (f).

The SEM pictures show that the cells are already attached on the titanium as well as on the zirconium dioxide specimen after 24 hours and built up cell formations. The epithelial cells with a polygonal shape lie flat on the two materials and form radial microvilli, which on the one side link to the material surface and on the other side present contact points towards the neighbouring cells (Figure 8). These formed cell layers can be also observed after 48 hours of culture on the zirkonium dioxide specimen (Figure 8f).

The SEM-images document the attachment and the development of complex structures on the tested materials by the cultured cells grown out of the tissue samples.

**Figure 9:**
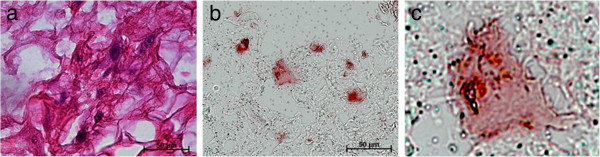
Cells in collagen membrane (a), HE staining (b), anti-cytokeratin detected cells in the collagen matrix (c), magnification of (b).

The cells seeded on the collagen membranes moved through the material and spread out within the collagen structure. With the aid of the different staining methods (HE and immunohistochemistry), the cells could be detected on the membranes surface and inside the samples (Figure 9).

## Discussion

Dental medicine is often concerned with the replacement and restoration of teeth. For these purposes different materials like titanium or zirconium dioxide are in common use, e.g. titanium for dental implants. Whereas Titanium is still the standard material of dental implants, Zirconia seems to be another good candidate that provides some aesthetic advantages like the white and teeth-like colour. Dental implants and other materials used in dentistry and maxillofacial surgery are in direct contact with the soft and hard tissue and numerous studies and *in vivo* experiments have focused on this topic. Most of them deal with the analysis of the osseointegration of the implants and therefore a lot of research is done with osteoblastic cells. In contrast, the present *in vitro* study highlights the first reactions of human gingival epithelium cells on the two implant materials, titanium and zirconium dioxide and additionally on a collagen membrane.

In current research, mainly two different methods for culturing keratinocytes *in vitro* are used. The direct explant technique applied here and the enzymatic method (cells are solved from the tissue, e.g. with trypsin, before cultivating). A third technique described by Lauer et al. 2001 [13] in which the explants are directly placed into the centre of the specimen was also used in our preliminary tests but showed a great variety concerning the resulting outgrowth of cells. This led us to use the more promising first method where the cells grow out the explanted pieces of gingival tissue.

This direct explant technique, described previously in other studies [13,14], showed continuous growth of phenotypic different cells derived from the human gingival biopsies of this study.

Preliminary tests in the run up of this study were done to compare the explant technique with the enzymatic technique. In the end the direct explant technique appeared to be more successful for culturing human gingival keratinocytes compared to the enzymatic method. Furthermore, this method required only small pieces of gingival tissue and generated higher cell amounts than the enzymatic method. These findings confirm with Kedjarune and co-workers [14]. The explant method was also used very successful by others [13,16]. When comparing the rate of outgrown cells using this method in the present study with the literature the results are very similar, stressing the reliability of this culture technique. 224 samples of the 242 tissue samples used here showed out growing epithelial cells. Only 18 of the samples (7%) showed no cell growth for unknown reason. In the case of 116 samples even an excrescence rate of 100% could be reached within this study.

With the explant technique the cells showed multi layered cell growth after 7–8 days in culture. This multi layered outgrowth with increasing thickness in the direction of the tissue shows strong similarities to the structure of the gingival epithelium *in vivo*.

The cells grown out of the tissue samples were characterized as gingival keratinocytes by immunohistochemistry staining with two established markers (anti-cytokeratin and anti-p63). The cells in this study expressed the typical keratin filamentous network in the cytoplasm of epithelial cells as described already by others [13,17-19].

The low-contrasted cells grown out of the tissue after 2–3 days were clearly detected by the used epithelial cell marker cytokeratin. Cells in this state of differentiation were also positive for p63, which is highly expressed in the basal layers of many epithelial tissues [20] and human epidermal stem cells [21]. A very low expression of this marker is shown by partially differentiated cells. Therefore the cultured cells of this study show basal cell characteristics, observed in the explant cultures as well as after sub cultivation. The results of the immunohistochemistry staining with anti-p63 and anti-cytokeratin documented that the cultivated cells showed typical characteristics of epithelial keratinocytes even over a long time in culture from 28 up to 70 days. In summary the cells grown out of the human gingival tissue of this study can be described as epithelial keratinocytes.

The two tested implant materials titanium and zirconium dioxide are both used as implant- and abutment material in dental implantology. These materials and their surface properties were also tested and evaluated mostly positive for implant and abutment applications by others [2,5,22-30].

The light and electron microscope images of the cultivated cells on the two implant material surfaces show that the outgrown cells attach to both materials to form associations after a culture period of only 24 hours. Typical multi layered cell complexes can also be observed after 24 and 48 h of culture, respectively.

The Richardson-stained cells on the tested materials show that the size and number of the emerging cellular layer is growing over time. This gives strong evidence for cell proliferation and migration. These results were achieved with a cell seeding density of 6 × 10^4^ cells/cm^2^. This is a very low seeding density compared with similar studies by others [13] who confirmed that a seeding density of up to 5 × 10^5^ cells per well (96-well plate, = 1,56 × 10^6^ cells/cm^2^) was necessary to ensure the adherence of the cultivated cells on different titanium surfaces (polished and plasma-sprayed) and to reach a confluent monolayer [13].

The cell seeding density of 6 × 10^4^ cells/cm^2^ in the present study was chosen to investigate and analyze the attachment and the cell-cell interaction of cells on the different test materials after short time periods up to 48 hours. Taking into account the very low cell seeding density used in this work, the structures of both surfaces, titanium and zirconia, seem to be beneficial for the growth, migration and proliferation of the epithelial cells in this study.

The electron microscopic images of the cells on the implant materials show that the cells attach very quickly on the surfaces. The cells form extensive appendices within 24 hours. These structures document a good adhesion to the surface and give also evidence for the formation of cell-cell contacts. The epithelial cells with a polygonal shape lie flat on both material surfaces described in specimen material. The results of the present study show that the cultivated gingival keratinocytes attached well on the two tested, structured titanium and zirconium dioxide surfaces after 24 and 48 hours. The growth, proliferation and migration of the cells were comparable to the cells being sub cultivated as control on the polystyrene surface. The surface structures of the titanium and zirconium specimens in this present study are therefore suitable for the application in the region of soft tissue. Such surface structures used e.g. for dental implants allows convenient cell attachment and proliferation. Other studies that also focus on the cell growth and attachment of gingival keratinocytes on different titanium surfaces describe more inconsistent results. For example Lauer et al. analysed cell characteristics on glossy polished (by mechanical process), sandblasted (the sandblasting was done with 250-μm aluminium oxide particles under a pressure of 2–3 bar) and plasma-sprayed titanium surfaces and two different cultivation techniques and got different results concerning cell activity depending on the treated surfaces [13].

The third material used in this study was Resodont collagen membrane. In dental implantology and maxillofacial surgery this material already finds its application e.g. as bone graft material or membrane for guided bone regeneration. Collagen is the major component of the extra cellular matrix and the basal membrane between connective tissue as well as the gingival epithelium. Furthermore, collagen is accumulated in the process of wound healing and plays therefore a decisive role in different important processes *in vivo* e.g. in the context of modern dentistry and implantology. The used membrane consists of (equine) collagen fibrils which are also suitable for the application in humans. In the present study, the ability of the cells to migrate, even against mechanical resistance, was evidenced by the experiments with the collagen membrane. The results show that this fibrous collagen material is permeable for epithelial cells. The fibrous, linked structure is beneficial for the use as a scaffold. Cells are able to grow along the material, spread between the collagen filaments and fill the spaces. Such cell growth and proliferation on collagen membranes was also represented by Glaum and others using this material for cultivating gingival keratinocytes [31,32]. Cell spreading and proliferation within the collagen membrane should allow the accelerated filling of defect sites by regenerated epithelial tissue in a three dimensional environment (*in-vivo* state), this corresponds to the findings of other groups working in this field [33,34].

## Conclusions

On the basis of the presented results of the accomplished *in vitro* tests, the collagen membrane seems promising as cell carrier or scaffold for implant purposes in epithelial cell tissue engineering like it is done in the context of bone reconstruction.

Numerous studies focused on the techniques for the *in vitro* cultivation of gingival keratinocytes and fibroblasts and the interaction between cells and bio- or implant-materials, respectively [12,13,16,35-38]. Our experiences with the *in vitro* cultivation of gingival keratinocytes have shown that the different treatment steps during the whole cultivation process (preparation, storage etc.) have major effects on the cultivation success especially compared to our experiences with other tissue derived cells, like the in vitro cultivation of osteoblast like cells [39]. Hence, it is of importance to emphasize the impossibility of comparing results of tests with different materials and methods.

The results of this study show that the chosen direct explant technique is appropriate for the successful preparation of human gingival keratinocytes. Additionally the used *in vitro* cultivation and sub-cultivation method suits for the application of the cells on different materials.

It could be shown that titanium, zirconium dioxide as well as collagen membrane specimen in this study allowed attachment and proliferation of the tissue derived cells, stressing the biocompatible properties of these different materials.

Human gingival derived cells are therefore a good candidate for *in vitro* biocompatibility-tests of implant materials, basic materials in prosthodontics as well as for other biomaterials used in the oral cavity.

## Competing interests

The authors declare that they have no competing interests.

## Authors’ contributions

All experiments were arranged and accomplished by JN under the supervision of BL and HPW. The final manuscript was read and approved by all authors. The whole project was supervised by HPW.
